# Development and evaluation of the SimArena Magdeburg (SAM): A randomized controlled trial of the impact of a low-cost 180° simulation arena for emergency medical simulation training on stress perception and the associated resuscitation quality in medical students

**DOI:** 10.3205/zma001784

**Published:** 2025-11-17

**Authors:** Niklas Leschowski, Hanno Brinkema, Sabine Darius, Juliane Wolter, Irina Böckelmann, Katrin Borucki, Dorothea Sauer, Rüdiger Christian Braun-Dullaeus

**Affiliations:** 1 Asklepios Clinic Wandsbek, Central Emergency Department, Hamburg, Germany; 2 Cologne-Merheim Medical Center, Department for Orthopedics, Plastic Surgery, Trauma Surgery, and Sports Traumatology, Cologne, Germany; 3 Otto-von-Guericke University Magdeburg, Faculty of Medicine, Occupational Medicine, Magdeburg, Germany; 4 Otto-von-Guericke University Magdeburg, Faculty of Medicine, Institute for Clinical Chemistry and Pathobiochemistry, Magdeburg, Germany; 5 University Hospital Magdeburg, Department of Cardiology and Angiology, Magdeburg, Germany

**Keywords:** simulation, simulated environment, simulation arena, medical education, emergency medicine, virtual reality

## Abstract

**Introduction:**

Simulation arenas, in which video recordings from multiple projectors are projected onto curved screens to increase the realism of emergency medical simulations, have so far been associated with high development costs of more than 100,000 euros. The objectives of this study were (1) to develop a cost-effective simulation arena, (2) to determine whether the simulation arena increases the realism of simulations and (3) whether it has an impact on the perception of stress and the associated quality of resuscitation.

**Methods:**

A pilot study was conducted to assess the level of satisfaction, realism, and perceived stress in two student courses using a questionnaire. In the randomized controlled SIMARENA trial, third to sixth year students were assessed for subjective stress and subjective resuscitation quality by a visual analogue scale (VAS), objective resuscitation quality by a resuscitation feedback system, and objective stress by cardiac reactivity.

**Results:**

The development costs of the SimArena Magdeburg (SAM) amounted to 7,726.50 euros. Almost all participants agreed that the SAM contributed to the perception of a realistic environment. There was a significant difference in perceived stress. Cardiac reactivity was also significantly higher when using the SAM. The subjective and objective resuscitation quality showed no significant difference.

**Conclusions:**

The SAM is the first low-cost 180° simulation arena that enables emergency medical simulations in a realistic, secure, quickly interchangeable, and standardized environment. It causes an increased stress perception without reducing the quality of resuscitation, providing an optimal learning environment in which stress levels can be tailored to the participants' learning objectives through various parameters.

## Zusammenfassung

**Einleitung:**

Simulationsarenen, in denen Videoaufnahmen von mehreren Projektoren auf maßgefertigte gebogene Leinwände projiziert werden, um die Realitätsnähe von notfallmedizinischen Simulationen zu erhöhen, waren bisher mit hohen Entwicklungskosten von über 100.000 Euro verbunden. Die Ziele dieses Forschungsprojektes waren (1) die Entwicklung einer kostengünstigen Simulationsarena, (2) die Untersuchung, ob die Simulationsarena die Realitätsnähe der Simulationen erhöht und (3) ob sie einen Einfluss auf das Stressempfinden und die damit verbundene Reanimationsqualität hat.

**Methoden:**

In einer Pilotstudie wurde im Rahmen zweier studentischer Lehrveranstaltungen anhand von Fragebögen die Zufriedenheit, die Realitätsnähe und das subjektive Stressempfinden untersucht. In der randomisierten kontrollierten SIMARENA-Studie wurde bei Studierenden des dritten bis sechsten Studienjahres das subjektive Stressempfinden und die subjektive Reanimationsqualität mittels einer visuellen Analogskala (VAS), die objektive Reanimationsqualität mittels eines Reanimations-Feedback-Systems und das objektive Stressempfinden mittels der kardialen Reaktivität untersucht.

**Ergebnisse:**

Die Entwicklungskosten der SimArena Magdeburg (SAM) beliefen sich auf 7.726,50 Euro. Fast alle Teilnehmenden waren sich einig, dass die SAM zur Wahrnehmung einer realistischen Umgebung beigetragen hat. Es gab einen signifikanten Unterschied beim subjektiven Stressempfinden. Auch die kardiale Reaktivität war bei Verwendung der SAM signifikant höher. Die subjektive und objektive Reanimationsqualität zeigte keinen signifikanten Unterschied.

**Schlussfolgerungen:**

Die SAM ist die erste kostengünstige 180° Simulationsarena, die notfallmedizinische Simulationen in einer realistischen, sicheren, schnell austauschbaren und standardisierten Umgebung ermöglicht. Sie erhöht das Stressempfinden, ohne die Reanimationsqualität zu beeinträchtigen und bietet eine optimale Lernumgebung, in der das Stressniveau durch verschiedene Parameter an die Lernziele der Teilnehmenden angepasst werden kann.

## 1. Introduction

Simulation arenas, that project videos from multiple projectors onto curved screens, to enable a realistic, quickly interchangeable, secure, and standardized environment representation for emergency medical simulations are rarely used in emergency medical education worldwide due to the high development costs, which can exceed 100,000 euros [1], [2], [3], [4], [5], [6], [7], [8], [9], [10]. The semicircular training rooms provide a protected and secure environment and allow the simulation of the wide range of locations and environmental conditions in which emergency medical personnel must operate, but where simulations are not regularly possible, such as busy roads, highways, railways, and even military battlefields [1], [2], [3], [4], [5], [6], [7], [8], [9], [10], [11], [12], [13]. Using video projection, environmental sounds, and flexible props, different scenarios can be quickly interchanged to create a standardized, realistic environment for learning and exams [1], [3], [7], [9], [10], [13]. In addition, environmental simulation can create realistic stress conditions, restrict the regular workflow and challenge the teamwork and interpersonal communication [9], [10], [13], [14], [15]. The high degree of realism in simulation training is of pertinence, as it aims to attain a high level of competence through the seamless integration of learned techniques into daily life and clinical situations [10], [16]. In addition, it can help develop a certain level of stress resistance. Processes such as communication, leadership, and decision-making under stress can be experienced in a targeted manner, while at the same time emphasizing the importance of complying with medical and non-medical safety standards [9], [10], [15]. There is evidence that stress can potentially reduce attention resources, increase distractibility, and impair resuscitation performance [17]. 

The overall objective of this research project was to develop the low-cost simulation arena “SimArena Magdeburg” (SAM) and to investigate the added value of simulation arenas, especially in comparison to frequent simulations in seminar rooms without environmental representation. As part of a pilot project, the satisfaction and subjective perception of the realism of the SAM's environment representation were to be investigated using a subjective questionnaire. Since studies have so far mainly used subjective questionnaires to investigate the effects of simulation arenas on participants in simulation training, a randomized controlled trial was designed to investigate how the use of SAM during a five-minute lay resuscitation affects the subjective and objective stress perception as well as the subjective and objective resuscitation quality of medical students. Our hypotheses were that the SAM would provide a satisfactory and realistic representation of the environment and that the subjective and objective stress levels of medical students during a 5-minute lay resuscitation would increase more and the subjective and objective resuscitation quality would decrease more with the SAM than without the SAM.

## 2. Methods

### 2.1. Development

**Figure 1:**
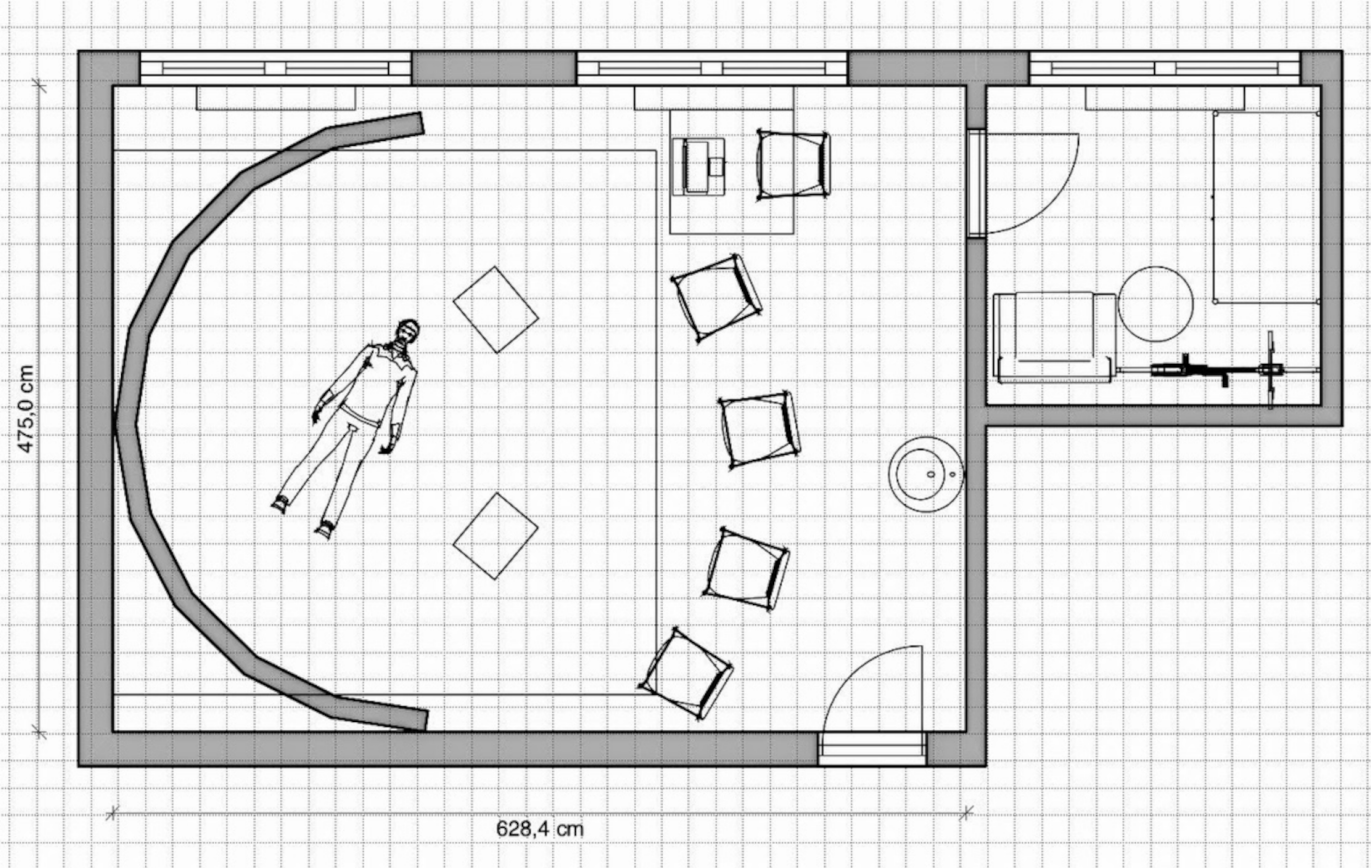
SAM floor plan (©Leschowski)

**Figure 2:**
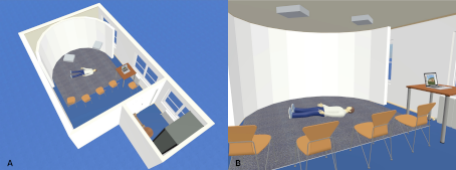
3D Model: (A) overview and (B) interior view of the SAM (©Leschowski)

**Figure 3:**
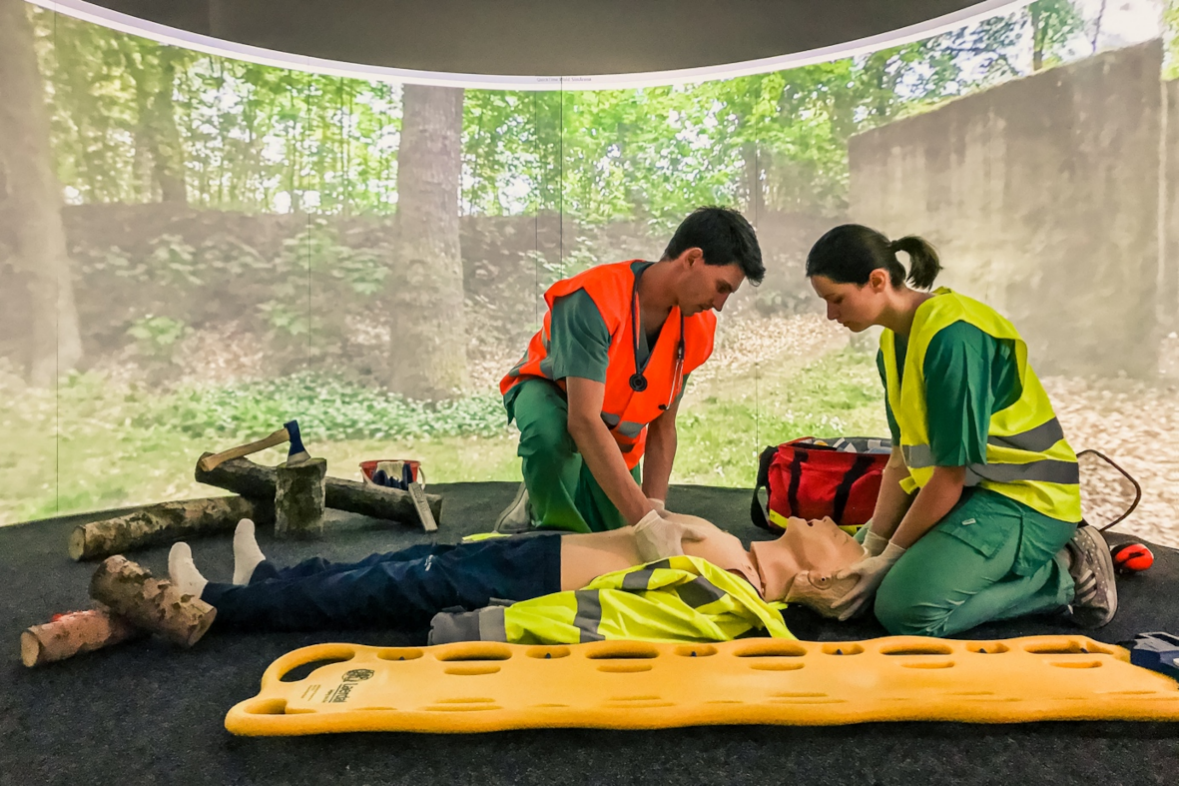
Forest scenario in SAM (©Leschowski)

The projection screen of the SAM is a semi-circular, 180° curved wooden construction consisting of several segments and coated with four millimeters of solid white paint. The short-throw projectors and ceiling mounts were placed so far away from the screen that both short-throw projectors illuminated all parts of the screen (see figure 1 (Fig. 1) and figure 2 (Fig. 2)). To combine the two independent projections of the short-throw projectors into a single projection, both short-throw projectors were synchronized using the NVIDIA Surround feature in the 3D Settings menu of the NVIDIA system control. The Immersive Display software allowed the projection to be adjusted to fit the curvature and dimensions of the projection surface (see figure 3 (Fig. 3)). For the panoramic video recordings, the Nikon KeyMission 360 4K was mounted on a tripod one meter above the ground and a five-minute recording was started. To convert the 360° panoramic video footage into 180° panoramic video footage, it was cropped to a horizontal angle of 180° using the video editing program iMovie. Additional sounds were added to the videos as needed. The video recordings can be adjusted to the size of the screen and played back using the QuickTime or VLC player. A dark gray carpet was installed to provide a color-coordinated background for the environment simulation and to make the participants’ knees more comfortable. In addition, a fan to simulate wind, a fog machine and a flash strobe were connected to the SAM via a radio switch set (see table 1 (Tab. 1)).

### 2.2. Pilot study

To investigate whether the goal of a realistic representation of the environment is achieved by the SAM and whether the SAM has an influence on the perception of stress the SAM was evaluated in a pilot study during two courses on polytrauma care at the Magdeburg Training Center for Basic Medical Skills (MAMBA Skillslab). Two simulation scenarios were performed in SAM environments and two simulation scenarios were performed in real-world environments not represented by the SAM. The SAM simulated a scene in a forest and a scene on a busy road. A stairwell and a street in front of the training center served as real environments. In all the simulations, participants had to care for a Laerdal Resusci Anne QCPR full body with airway head in a polytrauma scenario. Following the scenarios, all participants completed a questionnaire consisting of eight questions using Likert scales (see table 2 (Tab. 2)). Three questions were only added in the second course.

### 2.3. SIMARENA trial

#### 2.3.1. Subjective perception of stress

A visual analog scale (VAS-S) ranging from 0 mm (“no stress”) to 100 mm (“maximum stress”) served as the primary target measure of subjective stress perception. The secondary outcome measure was the State-Trait-Anxiety Inventory (STAI), which uses two questionnaires to distinguish between current anxiety, which varies in intensity over time and across situations (STAI-S), and habitual anxiety (STAI-T) [18]. The questionnaires each consist of 20 items on a four-point response scale. The values of the items are added together, resulting in two total scores between 20 and 80 [18]. The factors “current ability to act” (BEA), “emotional tension” (TEN), “emotional evaluation of the situation” (STIM), and “self-control” (SPAN) of the Nitsch Self-State Scale (EZ) were used as additional secondary outcome measures [19], [20]. The EZ consists of 40 characteristic words that are rated on a 6-point ordinal scale from 1 “hardly” to 6 “completely”. After converting the elementary values into area-transformed z'-values, these were summed and transformed into 14 binary factors in a 3-stage hierarchy [19], [20]. The scale of 1 to 9 is designed so that a higher value for each factor indicates a more positive subjective assessment of one’s ability to act [20]. The parameter “current motivation to act” (MOT) was also used to determine the motivation status of the test subjects prior to the study intervention [20].

#### 2.3.2. Objective perception of stress

Cardiac reactivity (CR), defined as the difference between the mean heart rate during resuscitation and a baseline period before resuscitation, was used as the primary objective measure of objective stress perception. For this purpose, continuous electrocardiography was performed using the Holter ECG medilog AR12plus (SCHILLER Medizintechnik GmbH, Obfelden, Switzerland). Using the medilog DARWIN2 software (SCHILLER Medizintechnik GmbH), electrical cardiac activity was automatically detected via three leads, verified, and incorrectly detected cardiac activity was manually corrected. The secondary outcome measure of objective stress perception was the heart rate (HR) and the heart rate variability (HRV) parameters SDNN (Standard Deviation of NN intervals), SNS Index (Sympathetic Nervous System Index), SI (Stress Index) and SD2 (Standard Deviation of the distances of the points from the major axis) during resuscitation. HRV analysis was performed using the Kubios HRV version 2.0 software (Biosignal Analysis and Medical Imaging Group, University of Kuopio, Finland). Respiratory rate (RR) was also used as a secondary outcome measure immediately after resuscitation and was continuously recorded by the Vernier Go Direct Respiratory Belt and analyzed using Vernier Graphical Analysis™ 4 software. Finally, the ratio of salivary alpha-amylase activity (sAA) immediately after resuscitation to a resting value before resuscitation was considered as another secondary outcome measure of objective stress perception. For this purpose, three saliva samples were collected from the subjects using saliva cups (SARSTEDT). The participants were instructed to place the saliva collection device's absorbent roll in one of their cheeks for two minutes without chewing or speaking [21], [22], [23]. After being stored at -20°C for a maximum of three days, the samples were subjected to automated in-vitro testing using the Roche Cobas6000 [23], [24], [25]. The samples were thawed at room temperature and centrifuged at 4000 revolutions per minute for ten minutes at room temperature. 10 μl of the sample were mixed with 990 μl of distilled water. After calibrating the measurement system with distilled water and a Roche calibration standard, the measurement was started using the c501 module in serum mode [24]. The Roche cobas c systems automatically calculate the analytical activity of the sample [24]. The conversion factor was: U/Lx0.0167=μkat/L. The kinetic method is based on the cleavage of 4,6-ethylidene-(G7)-1,4-nitrophenyl-(G1)-α,D-maltoheptaoside (Ethylidene Protected Substrate=EPS) by alpha-amylase, followed by the hydrolysis of all cleavage products using alpha-glucosidase (auxiliary enzyme) to p-nitrophenol (100% chromophore release) [24]. The color intensity of the formed p-nitrophenol was directly proportional to the alpha-amylase activity and was determined by measuring the increase in extinction [24].

#### 2.3.3. Subjective quality of the resuscitation

The primary target of subjective resuscitation quality was assessed using a visual analog scale ranging from 0 mm (“poor resuscitation”) to 100 mm (“excellent resuscitation”), which was completed by the subjects immediately after resuscitation.

#### 2.3.4. Objective quality of the resuscitation

The primary objective measure of the quality of resuscitation was the total score (TOTAL), which was calculated by the Laerdal Resusci Anne QCPR and the Laerdal SimPad PLUS SkillReporter immediately after resuscitation as an overall value of resuscitation performance based on the parameters of compression, ventilation, and time with CPR activity [26]. The algorithm used was developed by Laerdal Medical in close collaboration with members of the Emergency Cardiovascular Care (ECC) Subcommittee of the American Heart Association (AHA) and co-authors of the AHA Guidelines for Cardiopulmonary Resuscitation [27]. In addition, several secondary resuscitation parameters were also measured (see table 3 (Tab. 3)) [26]. As a further secondary objective, a resuscitation checklist was defined (see table 4 (Tab. 4)). This checklist was completed by the examiner during resuscitation and was based on the recommendations for lay resuscitation (“BLS procedure for adults”) of the 2015 guidelines of the German Resuscitation Council [28]. Unperformed actions were recorded as the number of errors (n).

#### 2.3.5. SIMARENA trial schedule

**Figure 4:**
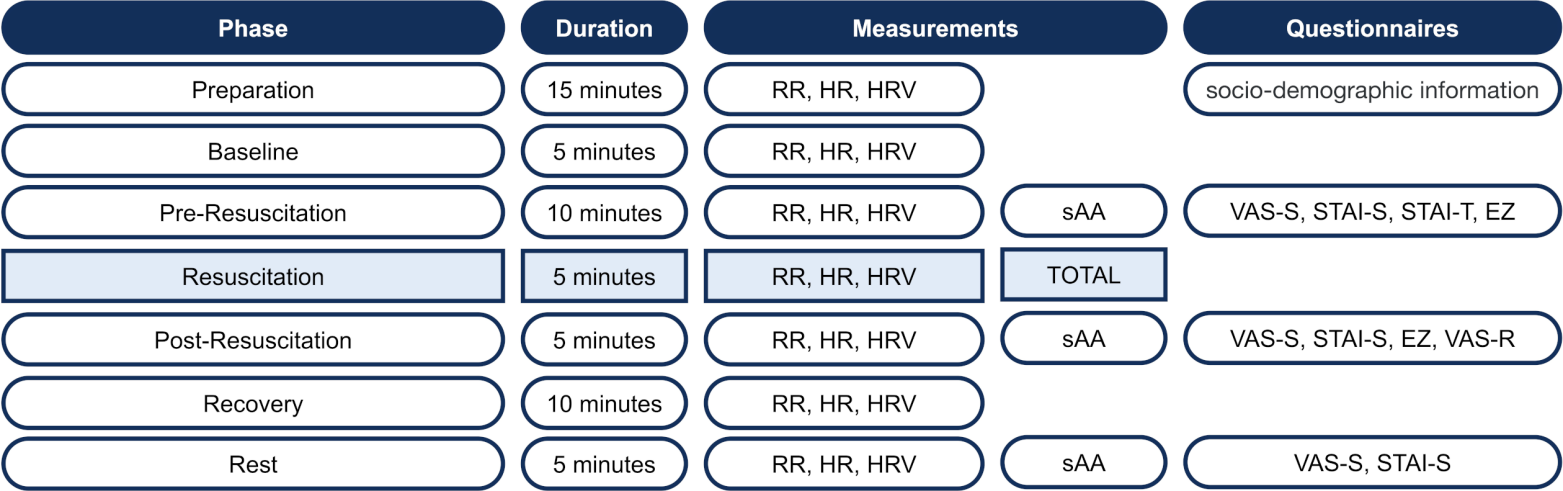
SIMARENA trial schedule (©Leschowski)

**Figure 5:**
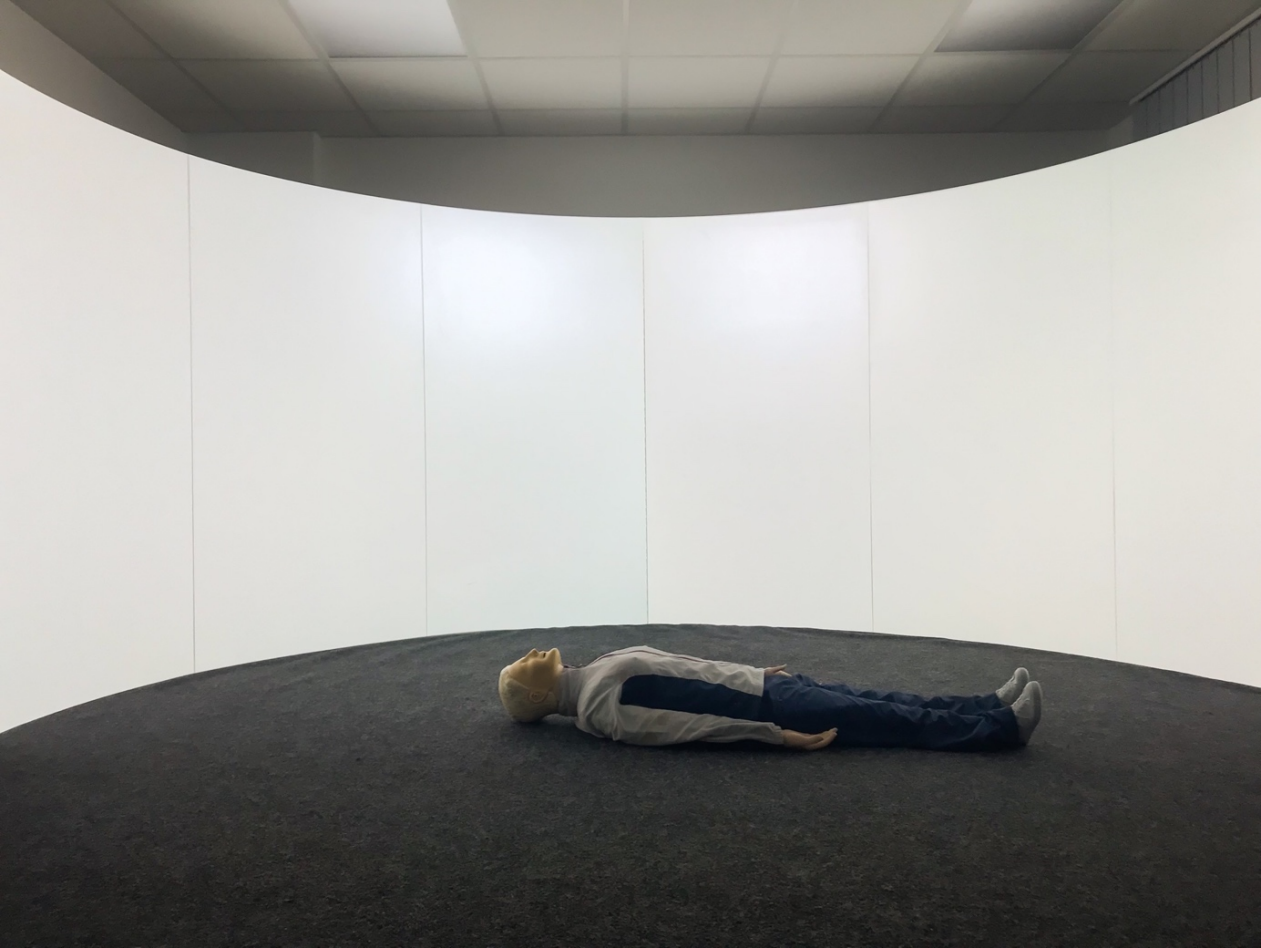
Control group simulation environment (©Leschowski)

**Figure 6:**
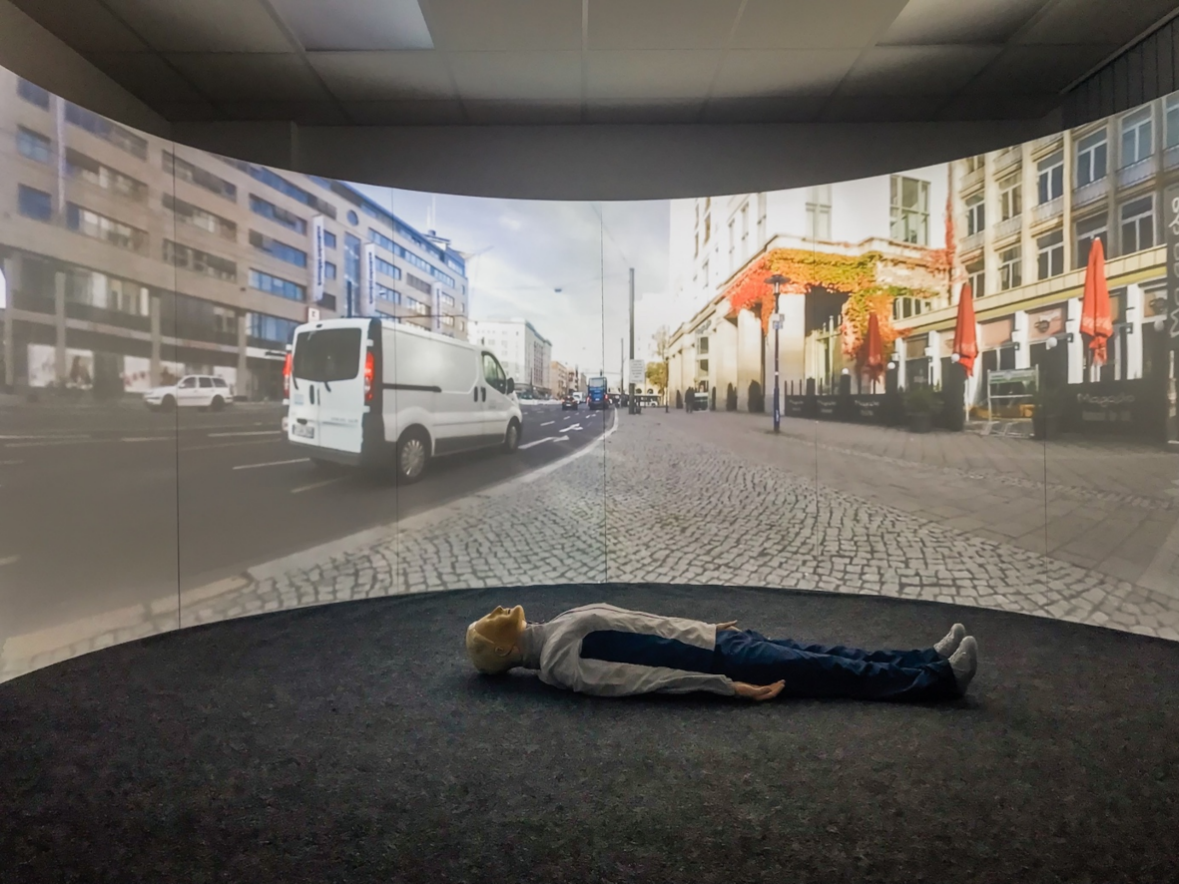
Intervention group simulation environment (©Leschowski)

The trial was divided into six phases (see figure 4 (Fig. 4)). Following a five-minute baseline phase, during which the participant was instructed to sit quietly on a chair and relax, the pre-resuscitation phase with the initial questionnaires and the first saliva sample commenced. It was instructed that the participant would find an unconscious person in the SAM and would perform a bystander resuscitation with ventilation after verifying the need for it. The participants in the control group (CG) entered the SAM, which displayed a white background without a tone signal (see figure 5 (Fig. 5)). Participants in the intervention group (IG) were shown a visual and auditory representation of a major road in the SAM (see figure 6 (Fig. 6)). If the participant wanted to call emergency services or request an automated external defibrillator, the examiner took over these tasks using a standardized response list (see table 5 (Tab. 5)). After five minutes, the resuscitation phase ended, and during the post-resuscitation phase the next questionnaires were completed, and a second saliva sample was collected. After five minutes, the ten-minute “recovery” phase was initiated, during which the participant was instructed to relax while sitting. During the “rest” phase, the participant was presented the final questionnaires, and a final saliva sample was taken.

### 2.4. Statistical analysis

A priori power analysis was conducted using G*Power 3: Statistical Power Analyses from Heinrich-Heine-University Düsseldorf based on a study by Mills et al. on the effects of emergency medical high- and low-fidelity simulations on cardiac reactivity [29]. To obtain statistically significant results regarding cardiac reactivity as the primary target of objective stress perception, a total sample of 38 participants would have been necessary based on these calculations. The participants were randomized into the intervention and control group using the RITA software (Randomization In Treatment Arms, StatSol, Lübeck) prior to the start of the trial. The statistical analysis was performed with the assistance of the Institute of Biometry and Medical Informatics using IBM SPSS Statistics 29 software (IBM, Ehningen, Germany). Descriptive analyses of the variables under investigation were conducted initially. The data was tested for normal distribution using the Shapiro-Wilk test. If the parameters were normally distributed, the Welch test was chosen as the metric test procedure. If the parameters did not follow a normal distribution, the Mann-Whitney U test was used as a non-parametric test. The distribution was tested using the Kolmogorov-Smirnov test for two samples after standardizing the data. The effect size was determined using Cohen’s d for metric methods and Pearson’s correlation coefficient r for non-metric methods.

## 3. Results

### 3.1. Pilot study

The SAM was constructed with development costs of 7,726.50 euros (see table 1 (Tab. 1)). All 38 participants completed the questionnaire in the survey. Most of the participants were third to fifth year students. The results of the survey demonstrate that the SAM contributed to the perception of a realistic environment through the video projection, ambient noise, and the props utilized. The video projections were predominantly perceived with peripheral vision, and subjective stress was reported during the simulations in the SAM (see table 2 (Tab. 2)).

### 3.2. SIMARENA trial

The SIMARENA trial involved 46 medical students in the clinical part of the study. 21 students were randomized to the intervention group (IG) and 25 to the control group (CG). Apart from the gender distribution, there were no significant group differences. More women than men were randomized to the intervention group (IG: 61.9%, CG: 44.0%) (see table 6 (Tab. 6)). A total of five HR (IG: 3, CG: 2) and six RR (IG: 3, CG: 3) recordings were excluded from the final analysis due to technical disturbances during the examinations or more than one percent of extrasystoles on the ECG [30], [31]. 

**Figure 7:**
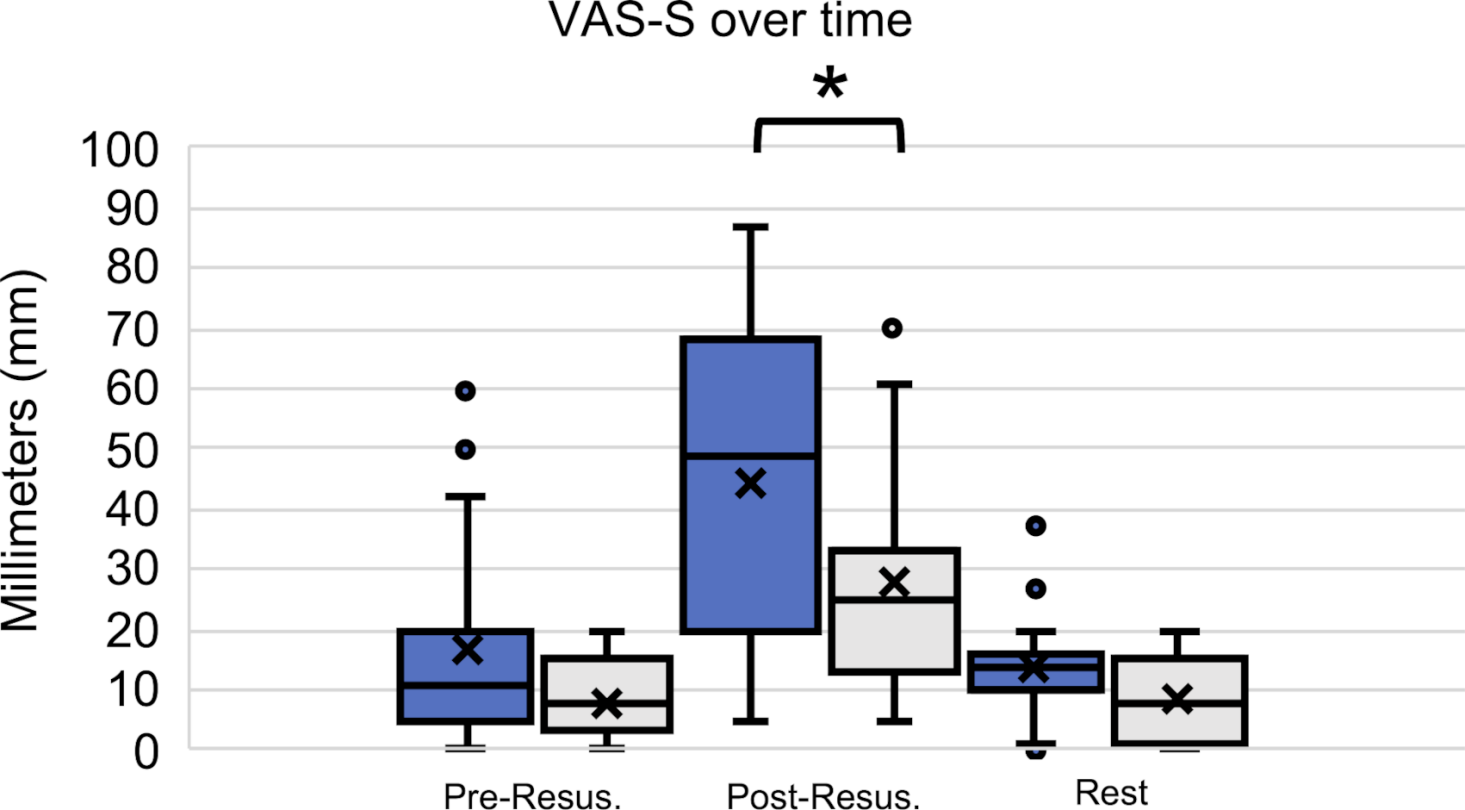
VAS-S over time (blue: IG, grey: CG)

**Figure 8:**
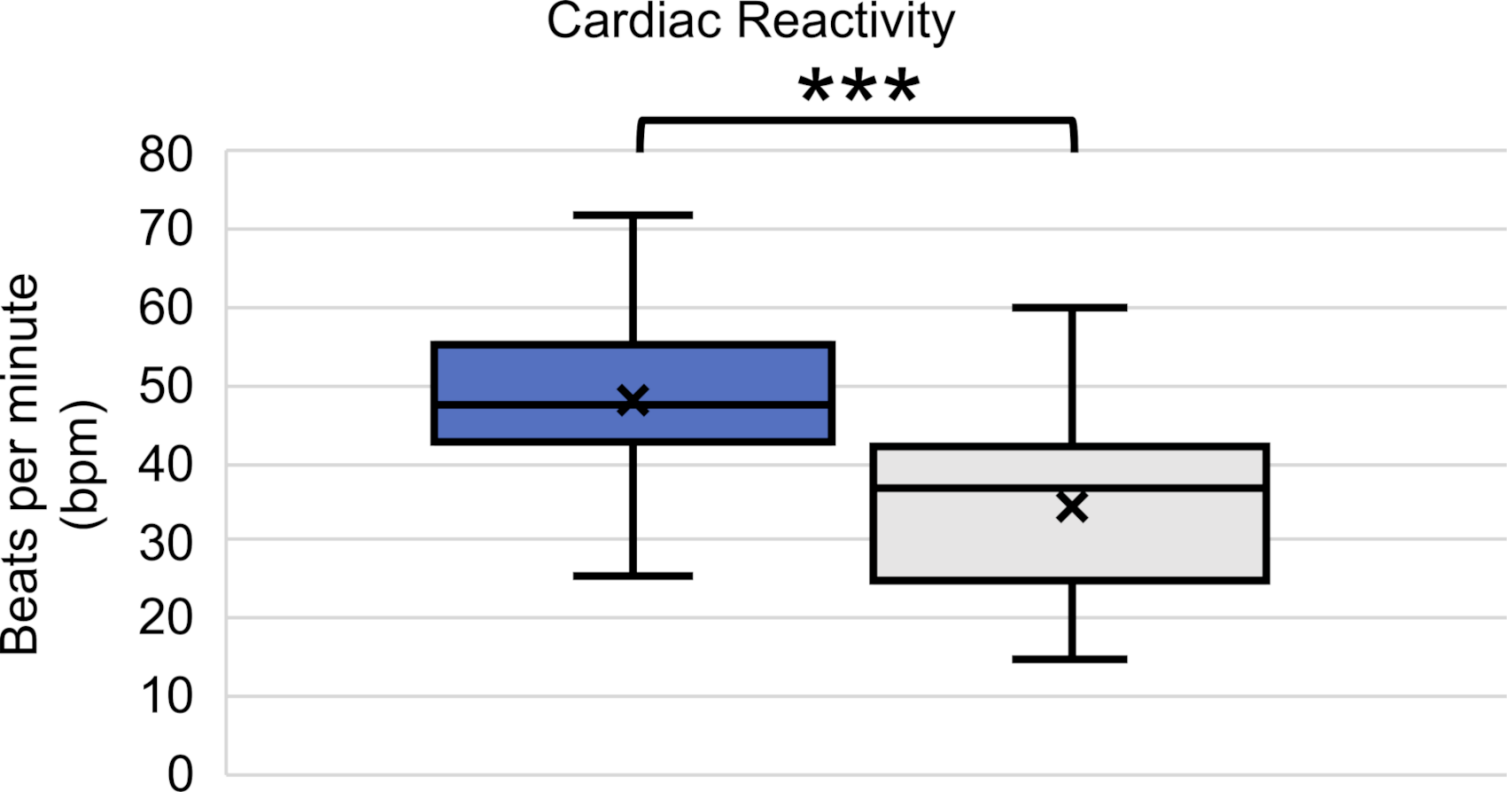
Cardiac reactivity (blue: IG, grey: CG)

**Figure 9:**
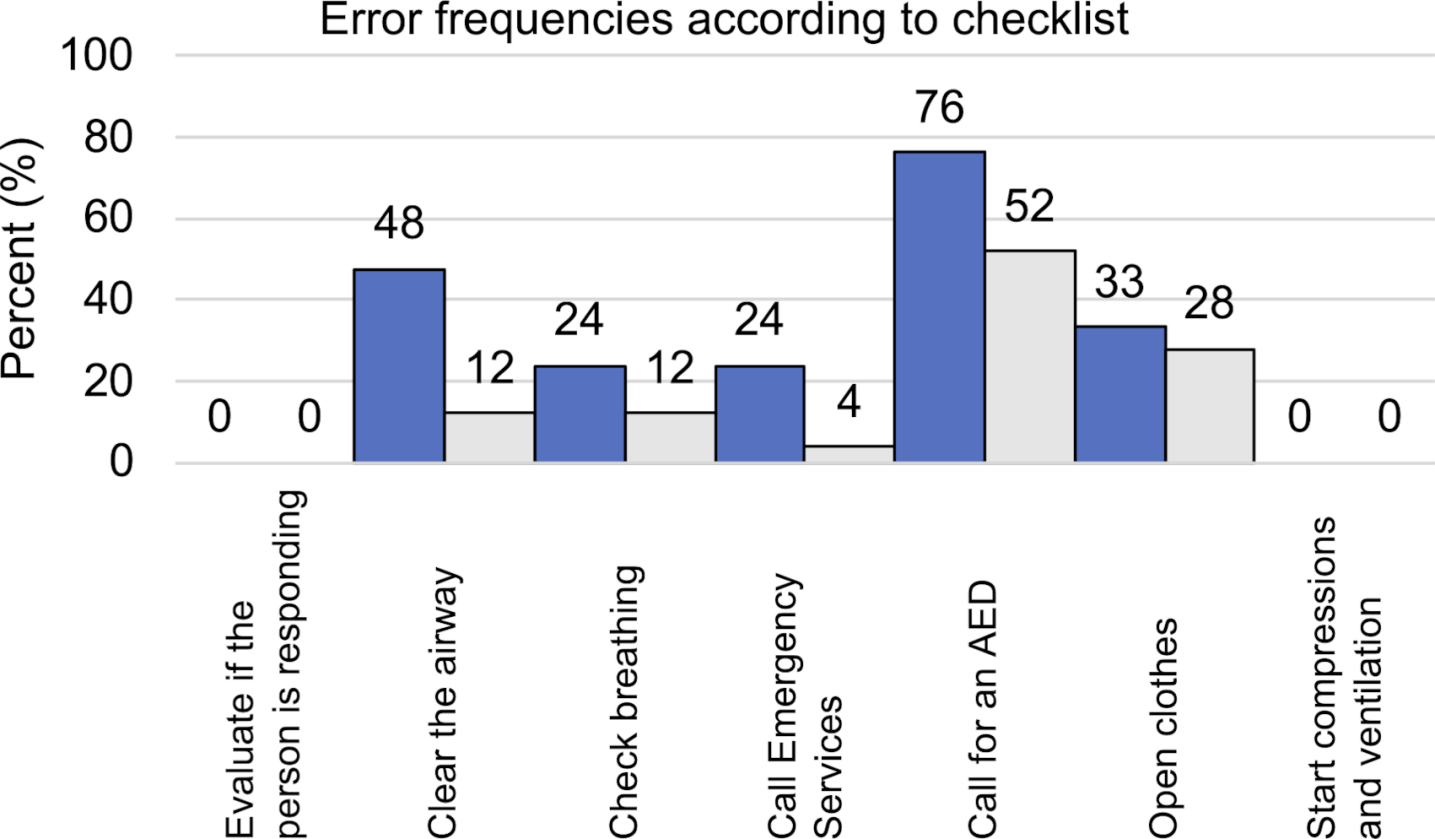
Error frequencies according to checklist (blue: IG, grey: CG)

The post-resuscitation VAS-S, as the primary outcome measure of subjective stress, showed a significant difference between IG and CG (see figure 7 (Fig. 7)). Also, the EZ parameters BEA, TEN, STIM and SPAN of the post resuscitation as secondary outcome measures showed significant differences between IG and CG. The secondary outcome measure STAI-S for post-resuscitation showed no significant difference between IG and CG (see table 7 (Tab. 7)). Cardiac reactivity, as the primary outcome measure of objective stress perception, showed a statistically significant difference between IG and CG (see figure 8 (Fig. 8)). The HR and HRV parameters SDNN, SNS, SI, and SD2, as well as the RR difference between the post-resuscitation- and baseline-phase and the RR of the post-resuscitation phase, also showed significant differences between IG and CG as secondary endpoints. The sAA ratio did not show a statistically significant difference between the IG and the CG (see table 8 (Tab. 8)). The VAS-R as the primary outcome measure of subjective quality of resuscitation and the TOTAL score as the primary outcome measure of objective resuscitation quality showed no significant difference between IG and CG (see table 3 (Tab. 3) and table 9 (Tab. 9)). The other resuscitation parameters also showed no significant differences, except for compressions with correct hand position and the number of errors according to the resuscitation checklist (see table 3 (Tab. 3) and figure 9 (Fig. 9)).

## 4. Discussion

### 4.1. Development of the SAM

With a development cost of 7,726.50 euros the SAM is the first low-cost simulation arena in Germany [7], [8]. However, due to the lower expenditure, the quality of the video recordings, video projections and ambient sounds will be lower [1], [2], [3], [5], [6], [7]. The diameter of the SAM is also slightly smaller than the diameter of comparable simulation arenas due to the spatial conditions [1], [5], [6], [7], [8], [10]. To provide enough space for the simulation, the SAM has a 180° projection screen instead of a 270° projection screen [1], [3], [4], [5], [7]. In the future, the quality of the environmental presentation could be further enhanced by using several parallel cameras to record the environment, a larger number of short-distance projectors, or several LCD monitors surrounding the simulation arena as screens, as well as stereoscopic three-dimensional projections. The integration of surround sound systems and scent generators has the potential to further enhance the immersive simulation experience. However, research into this topic is still pending [1], [2], [3], [4], [5], [6], [7], [8], [9], [10], [13], [32]. Unlike the arenas in Münster and Essen, developed with external partners, the SAM was built in-house and lacks an external technical contact [1], [2], [5], [6]. In the event that difficulties arise in adjusting the image to the SAM screen, assistance can be obtained by contacting the company Fly Elise-ng. Since its development, SAM has been utilized regularly for curricular and optional emergency medicine courses at Otto-von-Guericke University Magdeburg and Magdeburg University Hospital.

### 4.2. Pilot study

The survey results show a high level of student satisfaction with the SAM, that is also reflected in the ratings of comparable simulation arenas [7], [8], [9], [13]. The results of the survey also suggest that the SAM contributed to the perception of a realistic environment and stress. This is also described in other studies [7], [8], [9], [13]. Our hypothesis that the SAM would provide a satisfactory and realistic representation of the environment could thus be confirmed.

### 4.3. SIMARENA trial

#### 4.3.1. Subjective perception of stress

The significantly higher VAS-S values in the IG immediately after resuscitation suggests that the SAM's depiction of the environment contributes to an increase in subjective stress, an effect that has also been reported in comparable studies [7], [9], [13]. The significantly reduced parameters BEA, TEN, STIM, and SPAN of the EZ support this result. In contrast, the STAI-S showed no significant difference between IG and CG. In the literature, comparisons between high- and low-fidelity simulations often do not show significant differences in the STAI-S [17], [33]. In some studies, a significant increase in STAI-S was shown when additional stress was induced not through changes in virtual environmental conditions, but through changes in the behavior of actors or the perceived threat of injuries [34], [35], [36]. Our hypothesis that the subjective stress level of medical students increases more during a 5-minute lay resuscitation with SAM than without SAM could thus be confirmed.

#### 4.3.2. Objective perception of stress

The significant increase in CR as the primary target of objective stress perception suggests that SAM also objectively contributes to increasing stress perception. A significantly increased CR and HR can also be found in the literature [29], [34]. The significant changes in HRV and the significant increase in respiratory rate as secondary endpoints confirm this result. Tramer et al. have previously demonstrated a significant reduction in SDNN during psychological stress during resuscitation efforts [37]. It is imperative to acknowledge the disparity in gender distribution between the intervention and control group, as women generally exhibit higher heart rates compared to men. However, studies on HRV parameters have yielded contradictory results. Some studies have observed increased parasympathetic activity in women compared to men, while others have found increased sympathetic baseline activity in women [30], [31]. The ratio of sAA from pre- to post-resuscitation phase did not show a significant increase between the IG and CG. While some studies report similar results, others have found significant increases in sAA [22], [25], [38], [39], [40]. Due to large differences in mean values as well as partly negative results, however, a large interindividual variation in sAA must be assumed [21], [22], [38]. The sAA measurements may also have been influenced by physical exertion during resuscitation or the circadian rhythm of sAA [38], [41], [42]. Furthermore, it should be noted that there are different approaches in the literature regarding the storage and processing of saliva samples [21], [22], [23], [38], [40]. In summary, our hypothesis that the objective stress level of medical students increases more during a 5-minute lay resuscitation with SAM than without SAM was confirmed.

#### 4.3.3. Quality of the resuscitation

**Figure 10:**
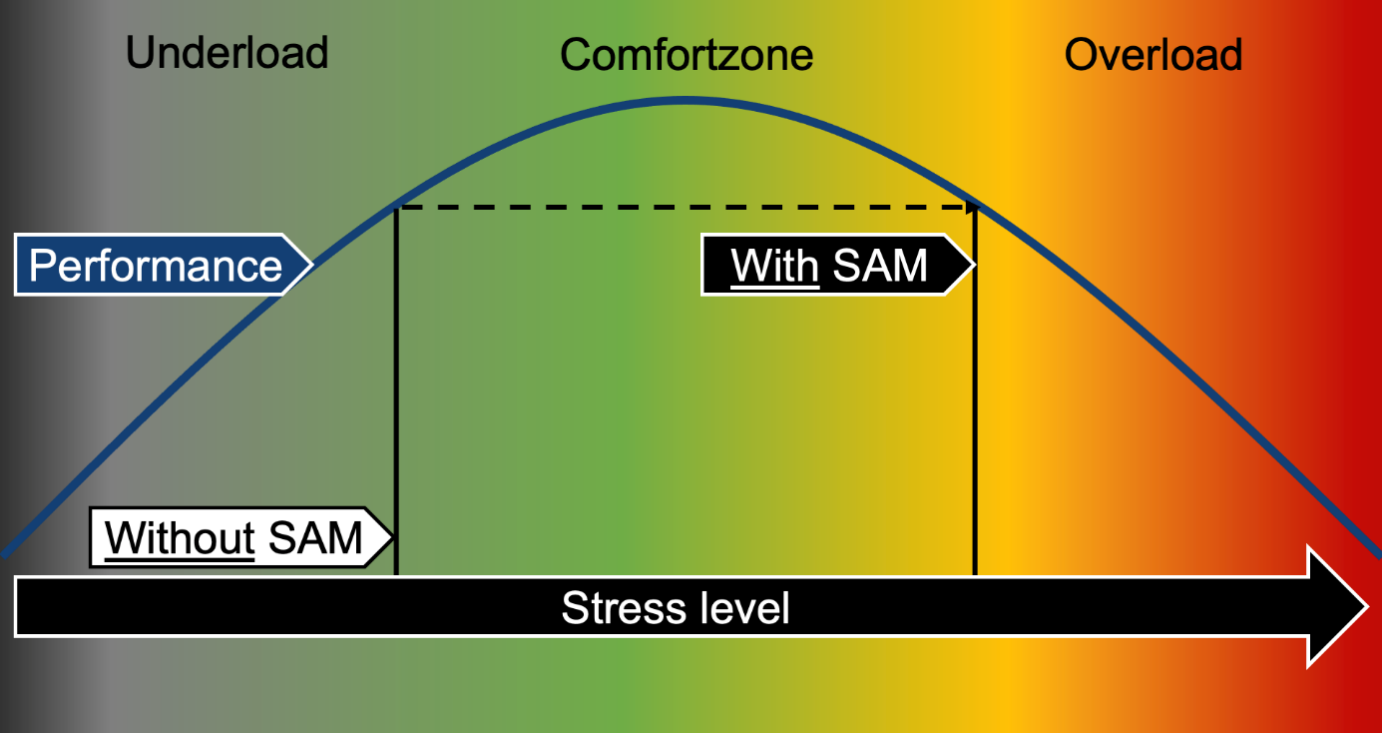
Influence of the SAM according to the Yerkes-Dodson theory

Given that the TOTAL-Score, VAS-R, and secondary endpoints did not demonstrate a statistically significant difference between the IG and CG, our hypothesis that the subjective and objective resuscitation quality of medical students decreases more during a 5-minute lay resuscitation with SAM than without SAM could therefore not be confirmed. However, the literature suggests that the quality of care deteriorates due to increased stress levels during simulations [9], [13], [17]. This discrepancy, with apparently increased subjective and objective stress levels in the IG without a corresponding reduction in resuscitation quality, can possibly be explained by the Yerkes-Dodson theory. The CG may have experienced the simulation without SAM in a state of understimulation with the same subjective and objective resuscitation quality as the IG, which was already in an incipient state of overstimulation on the Yerkes-Dodson curve due to the use of SAM (see figure 10 (Fig. 10)) [43]. The statistically significant reduction in compressions with correct hand position and the significantly increased mistakes in the IG as secondary outcome measures could be first indications of a reduced resuscitation quality in the context of the increased stress perception caused by the SAM. However, Wier et al. also demonstrated that aspects of team communication, team performance, and leadership were significantly reduced through simulation in simulation arenas, which were not investigated in the SIMARENA trial [9].

#### 4.3.4. Limitations

The significance of the results of the pilot study could have been increased by selecting comparable environments for the SAM environment representation and the real environments. Ultimately, the pilot study was only intended to answer whether the SAM subjectively generates a high level of satisfaction among the participants, whether a realistic environment can be represented and whether a simulation in the SAM induces stress. It should also be noted that the pilot study and the SIMARENA trial were not blinded. Overall, only a few students with varying levels of training voluntarily participated in the studies, indicating a high degree of preselection. Therefore, the results cannot be generalized to emergency medical personnel. Other video projections, changes in ambient noise, as well as the use of props, wind, and fog could have altered the stress perception and quality of resuscitation. Overall, the studies compare simulations without an additional environment representation with simulations with an environment representation using the SAM. This approach was chosen because it allows the additional benefit of a simulation arena to be investigated. 

## 5. Conclusion

The SAM is the first affordable 180° simulation arena in Germany that enables emergency medical simulations in a realistic, rapidly interchangeable, secure, and standardized environment. It increases stress levels without reducing the quality of resuscitation, providing an optimal learning environment in which stress levels can be tailored to the participants' learning objectives through various parameters.

## Authors’ ORCIDs

- Niklas Leschowski: [0000-0001-5319-7079]
- Sabine Darius: [0000-0002-8404-6406]
- Irina Böckelmann: [0000-0002-3905-3527]
- Katrin Borucki: [0000-0003-3648-2657]
- Rüdiger C. Braun-Dullaeus: [0000-0003-3888-6532]

## Acknowledgements

We would like to thank Dr. Winkler-Stuck, Korinna Wendt, the dean’s office, the commission for studies and education, and the student council for their support in the development and implementation of the SimArena Magdeburg.

## Competing interests

The authors declare that they have no competing interests. 

## Figures and Tables

**Table 1:**
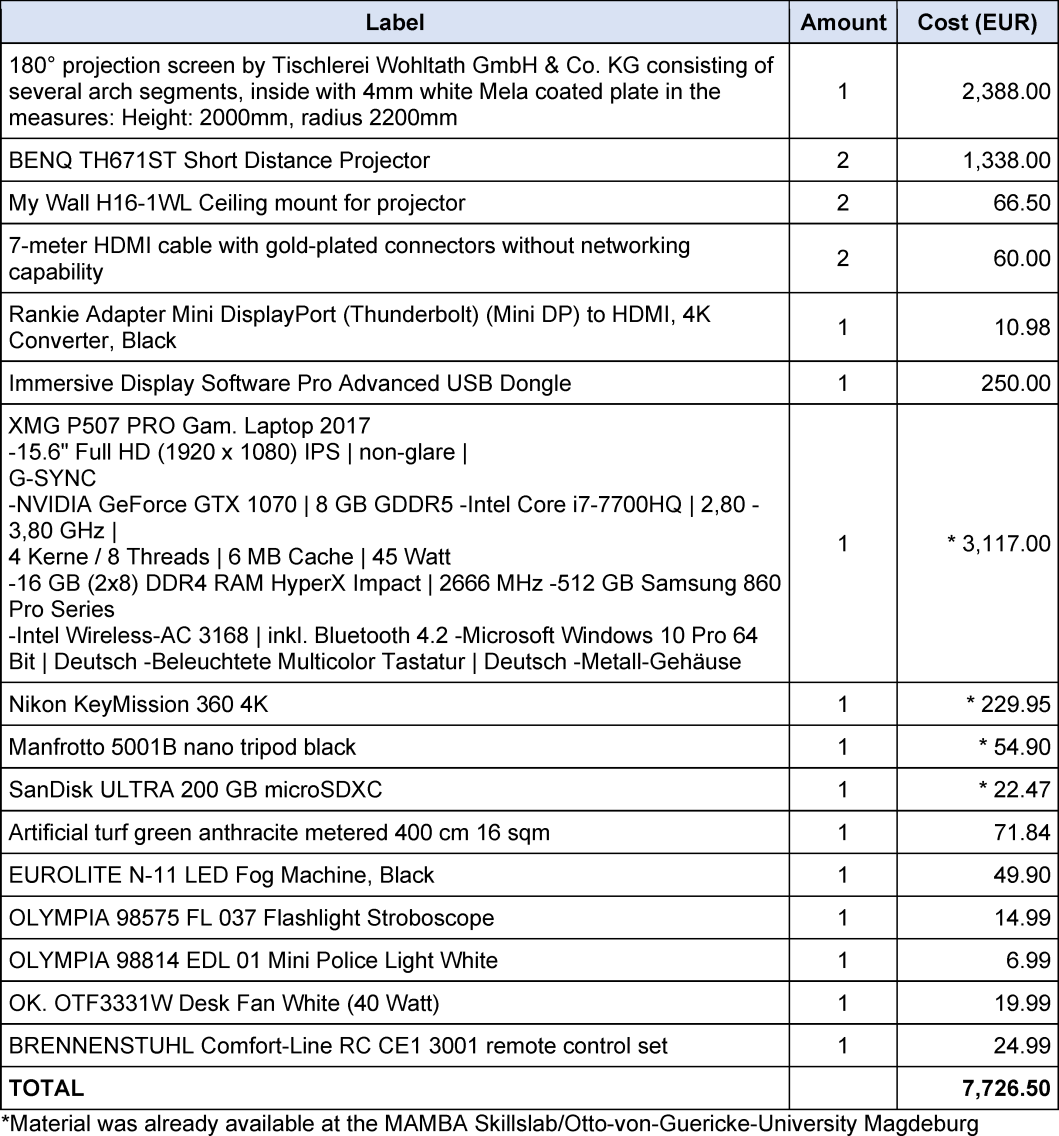
Material SimArena Magdeburg

**Table 2:**
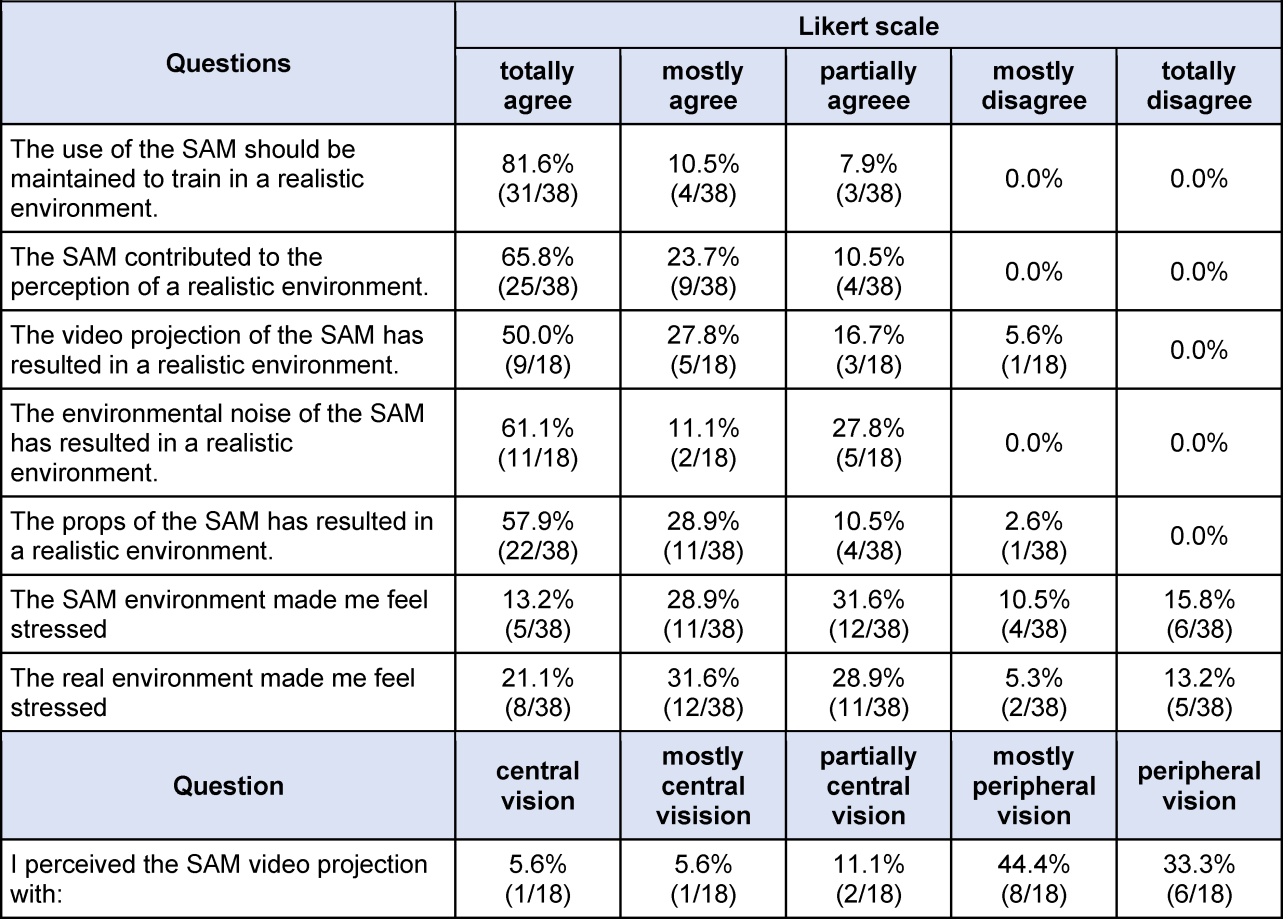
SAM survey

**Table 3:**
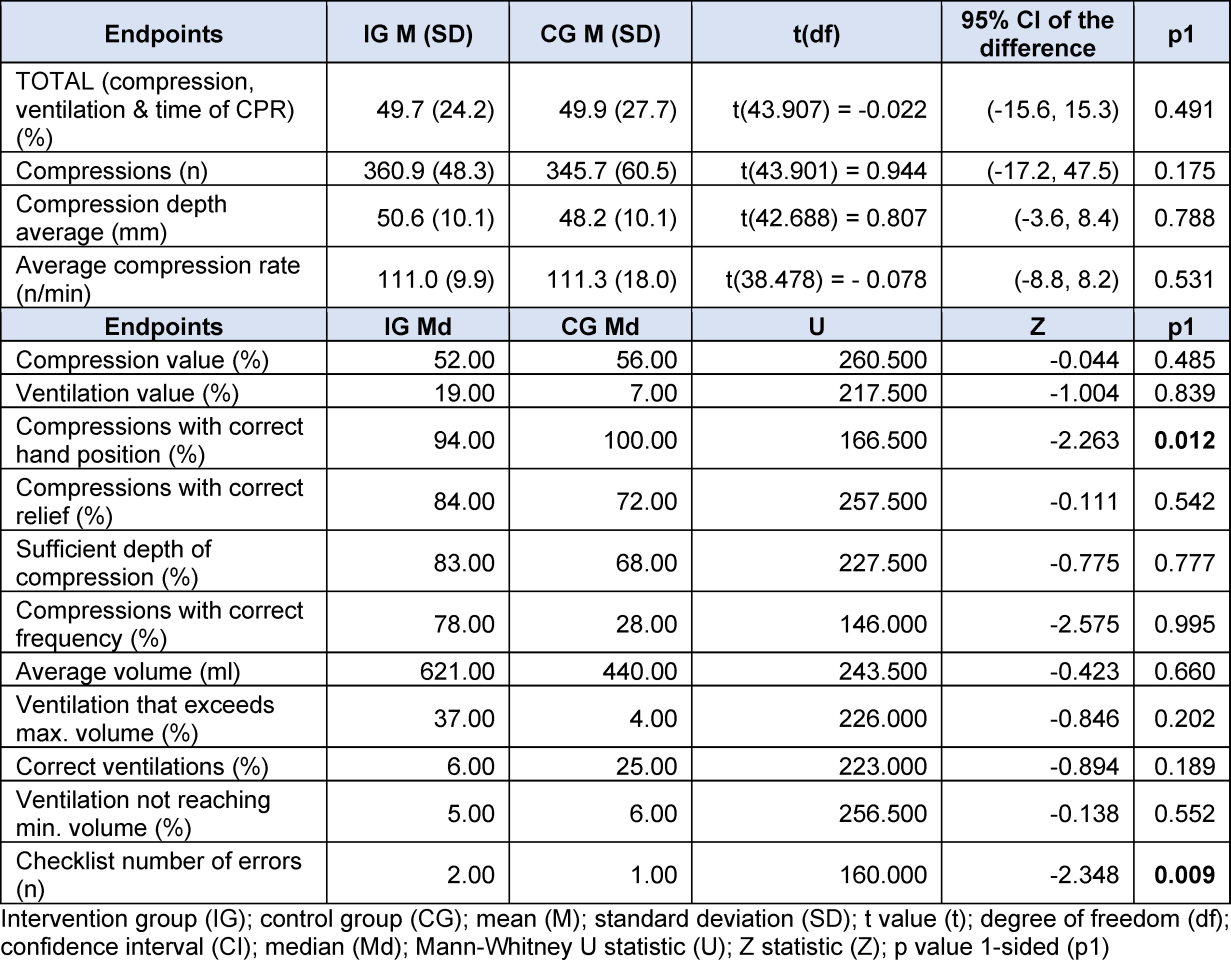
Results objective resuscitation quality

**Table 4:**
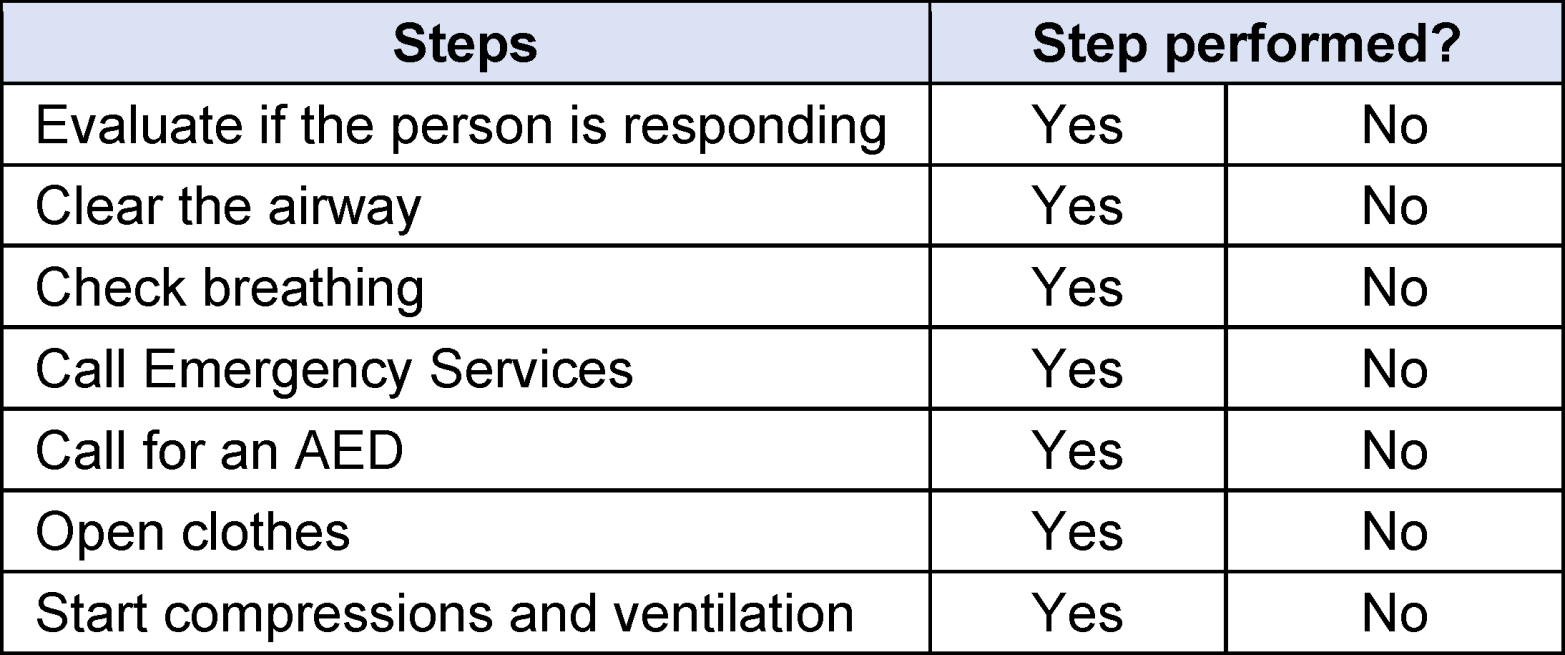
Lay resuscitation checklist

**Table 5:**
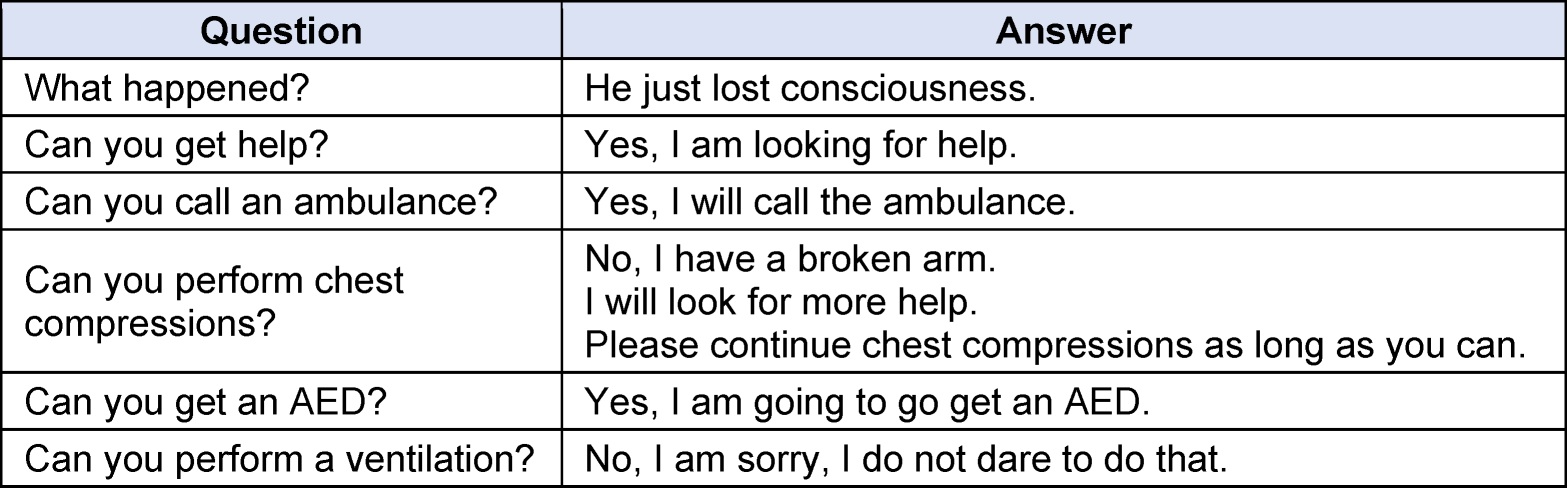
Standardized responses to subjects’ questions

**Table 6:**
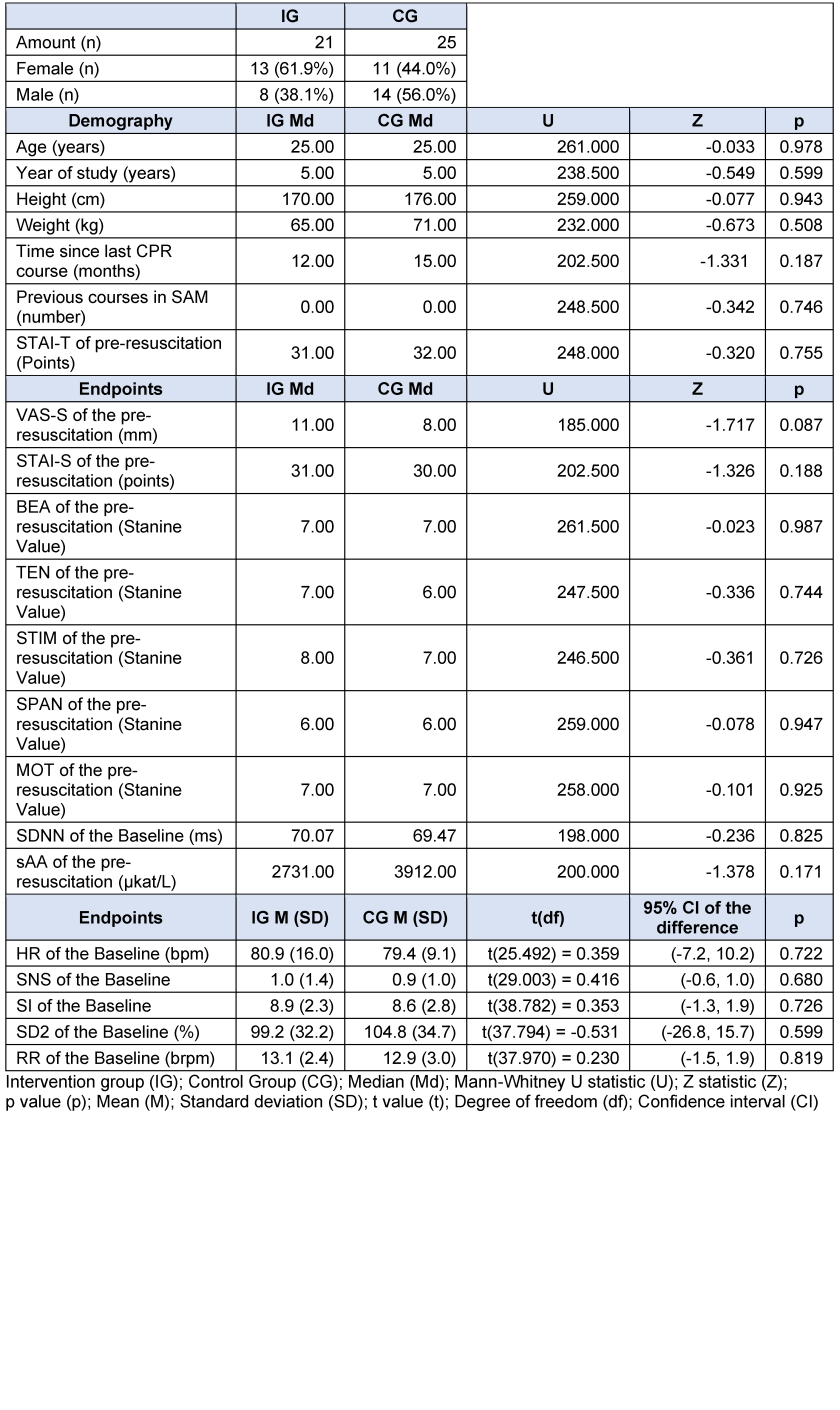
Sample description SIMARENA trial

**Table 7:**
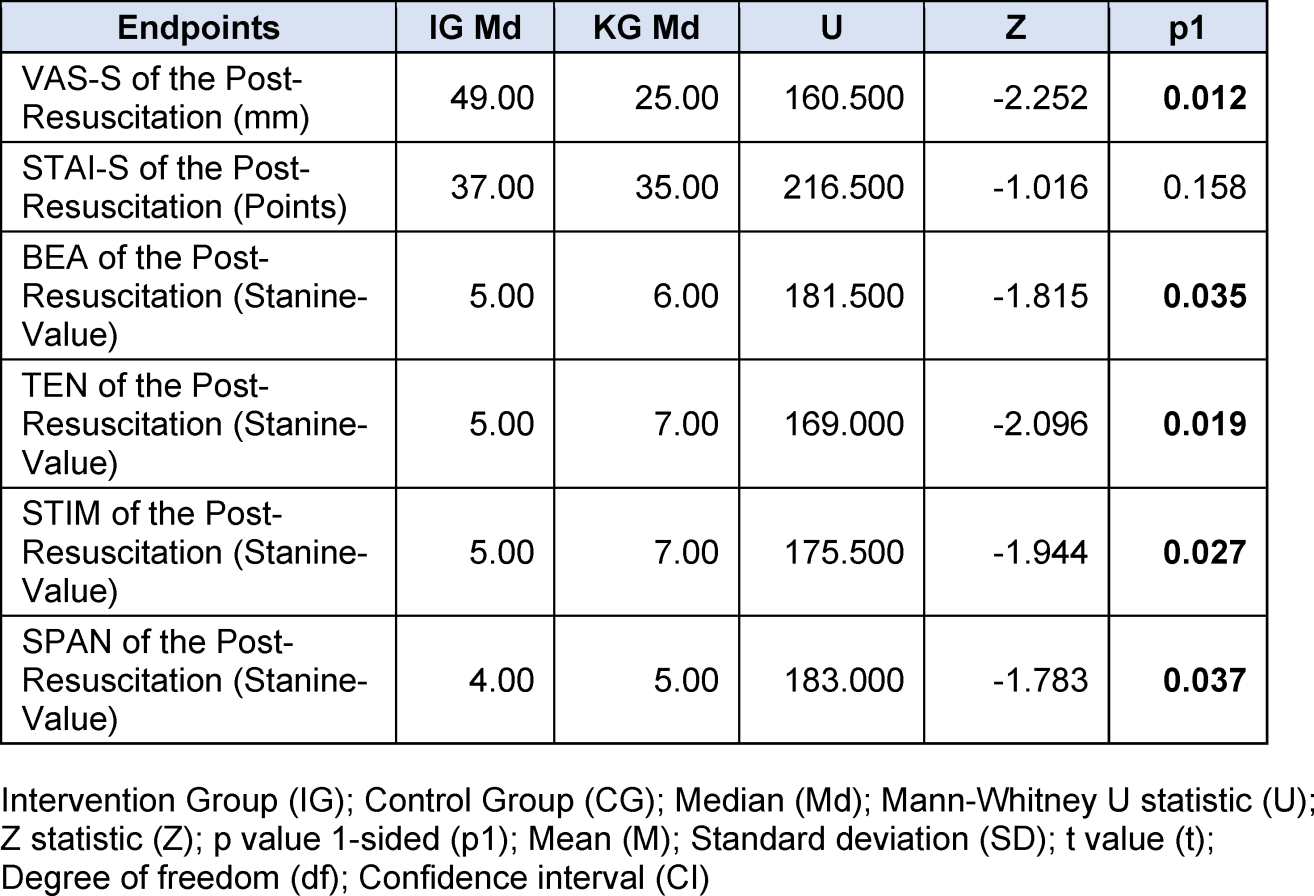
Results subjective stress perception

**Table 8:**
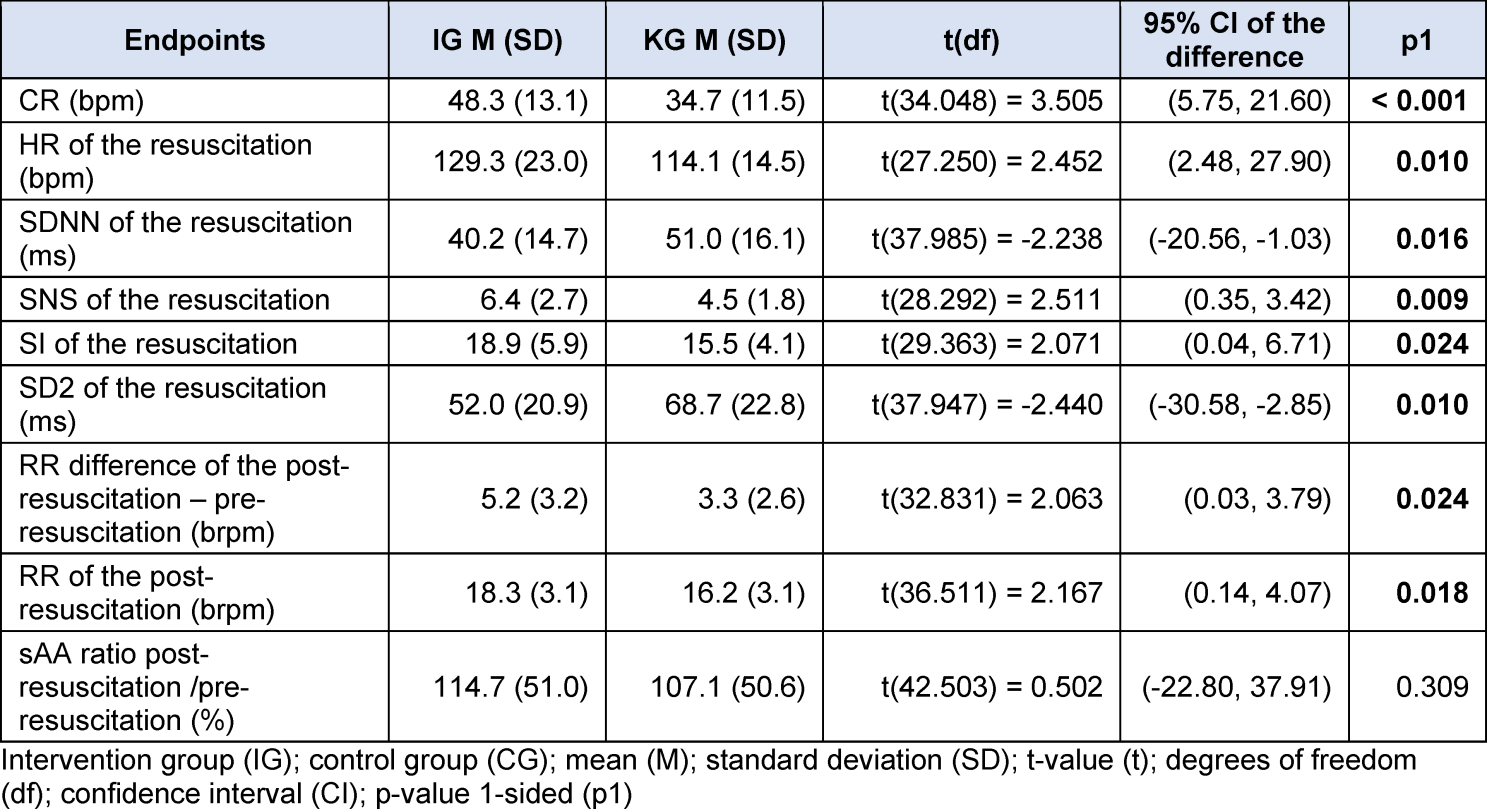
Results objective stress perception

**Table 9:**
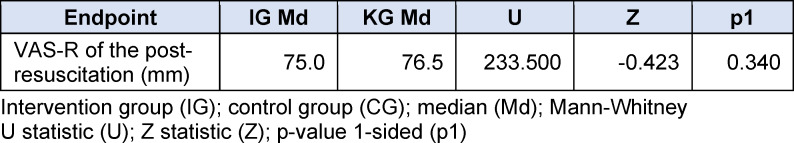
Results subjective resuscitation quality
